# HIV infection does not affect the risk of death of COVID-19 patients: A systematic review and meta-analysis of epidemiological studies

**DOI:** 10.7189/jogh.12.05036

**Published:** 2022-08-17

**Authors:** Giuliana Favara, Martina Barchitta, Andrea Maugeri, Giuseppina Faro, Antonella Agodi

**Affiliations:** Department of Medical and Surgical Sciences and Advanced Technologies “GF Ingrassia”, University of Catania, Catania, Italy

## Abstract

**Background:**

Even during the current Coronavirus Disease 2019 (COVID-19) pandemic, the infection with the Human Immunodeficiency Virus (HIV) continues to pose a major threat, worldwide. In fact, the World Health Organization (WHO) defined the HIV infection as a risk factor for both severe COVID-19, at hospital admission, and in-hospital mortality. Despite this evidence, however, there remains the need for investigating whether SARS-CoV-2 infection could increase the risk of death among people living with HIV (PLHIV). Thus, we conducted a systematic review and meta-analysis to assess the impact of the SARS-CoV-2 infection on the risk of death among PLHIV and HIV- seronegative people.

**Methods:**

The literature search was carried out on PubMed, Embase and Web of Science databases, from the inception to February 2022. Epidemiological studies on patients tested positive for SARS-CoV-2 infection, which compared the proportion of deaths between PLHIV and HIV-seronegative people, were considered eligible for the inclusion. The pooled odds ratio (OR) was obtained through meta-analysis of the comparison between PLHIV and HIV-seronegative people. Study quality was assessed by using the Newcastle-Ottawa Quality Assessment.

**Results:**

On a total of 1001 records obtained from the literature search, the present systematic review and meta-analysis included 28 studies on 168 531 PLHIV and 66 712 091 HIV-seronegative patients with SARS-CoV-2 infection. The meta-analysis showed no difference in the risk of death between PLHIV and HIV-seronegative patients (OR = 1.09; 95% confidence interval (CI) = 0.93-1.26; *P* > 0.001). However, a significant heterogeneity was found for this comparison (*I^2^* = 88.8%, *P* < 0.001).

**Conclusions:**

Although our meta-analysis suggests no difference in the risk of death of PLHIV with SARS-CoV-2 infection, if compared with HIV-seronegative patients, further research should be encouraged to improve the current knowledge about the impact of SARS-CoV-2 and HIV co-infection.

The Severe Acute Respiratory Syndrome Coronavirus 2 (SARS-CoV-2) pandemic has resulted in more than 3 million deaths worldwide [1], suggesting the need of more efforts in Public Health [2]. Major risk factors for Coronavirus Disease 2019 (COVID-19) adverse outcomes and mortality include preexisting chronic diseases (eg, diabetes, hypertension, obesity, and kidney diseases), as well as Human Immunodeficiency virus (HIV) infection [3-5]. Indeed, several findings suggest that the risk of COVID-19 mortality is more than two time higher among people living with HIV (PLHIV) than those HIV-seronegative [6-9].

Although the effect of many clinical conditions on the severity and outcomes of the COVID-19 is still on debate, HIV infection could suppress the immune system of PLHIV and make them at higher risk of SARS-CoV-2 infection and mortality. However, immunodeficiency of PLHIV could be a protective factor against the inflammatory response due to SARS-CoV-2 infection [10]. On July 15, 2021, the World Health Organization (WHO) stated that HIV infection is a risk factor for severe or critical COVID-19 at hospital admission and for in-hospital mortality [11,12]. This statement was based on a clinical surveillance of PLHIV in thirty-seven countries, regarding the risk of adverse outcomes after hospital admission for COVID-19. According to this report, the risk of developing severe or critical COVID-19 was ~ 1.3 times higher among PLHIV than those without it [11,12]. This was in part due to clinical conditions that are common in PLHIV and that put people at increased risk of severe disease and mortality. Moreover, the common use of antiretroviral drugs, mild immunodeficiency and chronic immune activation among PLHIV have been widely recognized as drivers of HIV complications [13,14].

Although preliminary studies found a similar prevalence of SARS-CoV-2 infection between PLHIV and HIV-seronegative people, there is still the need for expanding our knowledge about the impact of COVID-19 pandemic on the symptoms and clinical conditions of PLHIV [15-23]. In fact, it is important evaluating the impact of the SARS-CoV-2 infection on the risk of death and other clinical outcomes of PLHIV, so that these patients could be provided with more preventive strategies and Public Health policies [15,16,24-30]. Previous meta-analyses reported controversial results about the association between HIV infection and the risk of death among COVID-19 patients [31,32].

For all these reasons, the hypothesis that SARS-CoV-2 infection could increase the risk of death among PLHIV should still be investigated and confirmed by pooling data from previous studies. Thus, we have conducted a systematic review and meta-analysis of epidemiological studies evaluating the impact of SARS-CoV-2 infection on the risk of death among PLHIV and HIV- seronegative people.

## METHODS

### Literature search

A systematic literature search was carried out using the following terms: (COVID-19 or SARS-CoV-2 or Novel Coronavirus) AND (HIV or Human Immunodeficiency Virus or AIDS or Acquired Immune Deficiency Syndrome). The methodology of the current systematic review was in line with the Preferred Reporting Items for Systematic Reviews and Meta-analyses (PRISMA) statements and the Cochrane Handbook’s guidelines [33] (PRISMA checklist available in the File S1 in the **Online Supplementary Document**). Two of the authors (GF, GF) independently carried out a literature search of articles indexed in the PubMed, Embase and Web of Science databases, from their inception to February 2022. According to the PICO framework (File S2 in the **Online Supplementary Document**), the following selection criteria had to be meet: (i) epidemiological studies (ii) on patients tested positive for SARS-CoV-2 infection (iii) which compared the proportion of deaths between PLHIV and HIV-seronegative people. By contrast, the following documents were excluded: (i) abstracts without full text and non-English articles; (ii) case reports or case series; (iii) studies not including a group of patients with the coinfection; (iv) comments, letters, editorials, and reviews.

After removing duplicates, two authors independently screened titles and abstracts of retrieved articles according to the above- mentioned criteria. All eligible articles were full-text reviewed to evaluate whether selection criteria were fully met. The reference list of selected articles was checked to search for further relevant articles and any controversy was resolved by consulting a third author (AA). The following information were extracted from all the included studies: first author, year of publication, country, study design, age, and number of deaths in PLHIV and HIV-seronegative patients.

### Quality assessment

Study quality was assessed by two authors (GF and GF) using the Newcastle-Ottawa Quality Assessment Scale, which evaluated potential bias related to patient selection, comparability, and outcome. The potential existence of publication bias was assessed by visual inspection of the Funnel plot and by performing the Egger’s test.

### Statistical analyses

The main outcome of our meta-analysis was the risk of death of PLHIV if compared to HIV-seronegative patients. Thus, we used the number of deaths in each group to calculate, when not reported in the original article, the odds ratio (OR) with 95% confidence interval (95%CI) associated with the risk of death. Pooled estimate was obtained through meta-analysis and reported as the OR for the comparison of PLHIV with HIV-seronegative patients. Heterogeneity across studies was tested and measured using the Q-statistics and the *I^2^* index, respectively. In presence of significant heterogeneity (*P* for Q-statistics <0.1 and *I^2^*>50%), a random effect model was applied. Statistical analyses were performed using STATA (version 17).

## RESULTS

**Figure 1:**
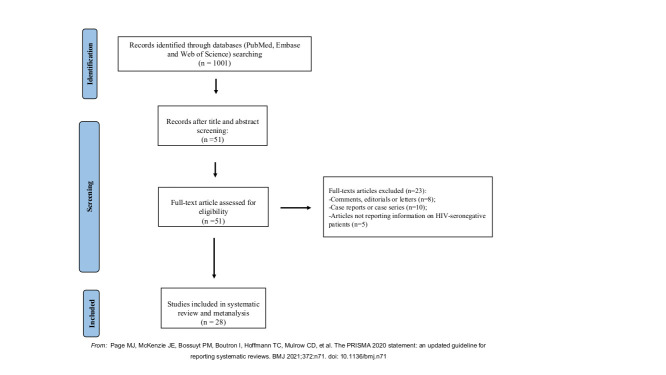
PRISMA flow diagram of study selection.

The PRISMA flow diagram reported in **Figure 1** shows the selection of studies included in the present systematic review. A total of 1001 records were obtained from the literature search, of which 11 were initially excluded because not providing the full-text. In addition, we excluded 939 after screening their titles and abstracts. Thus, 51 articles were subjected to full-text screening and 23 were excluded according to exclusion criteria. In particular, 8 were comments, editorials or letters to the editor, 10 were case reports or case series, and 5 did not report information on HIV-seronegative patients. Therefore, a total of 28 articles are included in the present systematic review and meta-analysis, and their main characteristics are shown in **Table 1**. All the included studies were published in years between 2020 and 2021, of which 15 were conducted in USA, 4 in United Kingdom, 2 in Spain, 4 in South Africa, 1 in Chile, 1 in Israel, and 1 in Iran. Overall, the sample size of PLHIV and coinfected with SARS-CoV-2 ranged from 4 to 92 643, while the sample size of HIV-seronegative people ranged from 23 to 45 565 024. Moreover, 26 were retrospective studies and 2 were prospective studies. With respect to the primary outcome, all the studies included compared number deaths due to SARS-CoV-2 infection in PLHIV and HIV-seronegative patients.

**Table 1:** Characteristics of studies included in the systematic review

Study	Year	Country	Study design	Age (years)	HIV+		HIV-		
**Death**	**Total**	**Death**	**Total**	**Quality of literature**
Flannery et al. 2021 [34]	2021	United States	Retrospective	Mean = 58.3 (SD = 12.4) among PLHIV; Mean = 64.3 (SD = 16.8) among HIV- seronegative	25	99	2703	10 103	7
Yang et al. 2021 [35]	2021	United States	Retrospective	Median = 49 (IQR = 36-60) among PLHIV; Median = 47 (IQR = 32-61) among HIV – seronegative	445	13 170	25 685	1 423 452	7
Hadi et al. 2020 [36]	2020	United States	Retrospective	Mean = 48.2 (SD = 14.2) among PLHIV; Mean = 48.8 (SD = 19.2) among HIV – seronegative	20	404	1585	49 763	5
Ceballos et al. 2021 [9]	2021	Chile	Prospective	Median = 44 (IQR = 26-85) on the overall population	5	36	4360	18 285	7
Lee et al. 2021 [37]	2021	United Kingdom	Retrospective	Median = 57 (IQR = 50-63) among PLHIV; Median = 56 (IQR = 51-62) among HIV sero-negative	13	68	35	181	6
Venturas et al. 2021 [38]	2021	South Africa	Retrospective	Median = 45 (IQR = 38-56) among PLHIV; Median = 52.5 (IQR = 39.8-61) among HIV sero-negative	16	108	54	276	7
Braunstein et al. 2020 [39]	2020	United States	Retrospective	NA	312	2410	16 160	202 012	5
Diez et al. 2021 [40]	2021	Spain	Retrospective	Median = 53 (IQR = 46-56) on the overall population	2	45	12	105	4
Cabello et al. 2021 [41]	2021	Spain	Retrospective	Median = 46 (IQR = 37-56) on the overall population	1	31	903	7030	6
Brown et al. 2020 [42]	2020	England	Retrospective	Median = 60 (IQR = 51-72) among PLHIV; Median = 83 (IQR = 74-89) among HIV sero-negative	99	92 643	49 483	45 565 024	4
Tesoriero et al. 2020 [43]	2020	United States	Retrospective	Mean = 54 (SD = 13.3) among PLHIV	207	2988	14 522	374 257	6
Chang et al. 2021 [44]	2021	United States	Retrospective	Mean = 54 (SD = 10) among PLHIV; Mean = 58 (SD = 25) among HIV sero-negative	1	61	223	12 921	6
Gudipati et al. 2020 [45]	2020	United States	Retrospective	Median = 51 among PLHIV; Median = 52 among HIV sero-negative	23	278	5919	64 993	4
Parker et al. 2020 [46]	2020	South Africa	Retrospective	Mean = 46.2 among PLHIV; Mean = 49.1 among HIV sero-negative	6	24	22	89	4
Kaplan-Lewis et al. 2021 [47]	2021	United States	Retrospective	Median = 56.8 (IQR = 18.2-79.4) among PLHIV; Median = 57.8 (19.4-91.7)	10	110	37	194	6
Bhaskaran et al. 2021 [7]	2021	United Kingdom	Retrospective	Median = 48 (IQR = 40-55) among PLHIV; Median = 49 (34-64) among HIV-seronegative	25	27 480	14 857	17 255 425	6
Geretti et al. 2020 [48]	2020	England, Scotland, and Wales	Prospective	Median = 56 (IQR = 49-62) among PLHIV; Median = 74 (IQR = 60-84) among HIV sero-negative	30	122	13 969	47 470	6
Boulle et al. 2020 [6]	2020	South Africa	Retrospective	>20 on the overall population	115	3978	510	18 330	6
Jassat et al. 2021 [49]	2021	South Africa	Retrospective	Median = 54 (IQR = 40-66) on the overall population	3407	13 793	30 697	137 986	5
Miyashita et al. 2021 [50]	2021	United States	Retrospective	NA	23	161	1235	8751	5
Sun et al. 2021 [51]	2021	United States	Retrospective	Median = 50 (IQR = 36-59) among PLHIV; Median = 47 (IQR = 32-61) among HIV sero-negative	196	8270	23 831	1 426 984	6
Nagarakanti et al. 2021 [52]	2021	Israel	Retrospective	Median = 59 (IQR = 51-67) among PLHIV; Median = 49 (IQR = 41-73) among HIV sero-negative	3	23	6	23	5
Rosenthal et al. 2020 [53]	2020	United States	Retrospective	Median = 57 (IQR = 41-71) on the overall population	37	252	7318	64 529	4
Sigel et al. 2020 [16]	2020	United States	Retrospective	Median = 61 (IQR = 54-67) among PLHIV; Median = 60 (IQR = 55-67) among HIV sero-negative	18	88	81	405	6
Stoeckle et al. 2020 [54]	2020	United States	Retrospective	Median = 60.5 (IQR = 56.6-70) among PLHIV; Median = 60.5 (IQR = 56.6-70) among HIV sero-negative	2	30	14	90	5
Durstenfeld et al. 2021[55]	2021	United States	Retrospective	Mean = 56 (SD = 13) among PLHIV; Mean = 62.3 (SD = 17.9) among HIV sero-negative	36	220	3290	21 308	5
Esfahanian et al. 2021[56]	2021	Iran	Retrospective	NA	4	4	151	496	4
Yendewa et al. 2021 [57]	2021	United States	Retrospective	Mean = 48.34 (SD = 13.59) on the overall population	46	1635	61	1609	7

SD – standard deviation, PLHIV – people living with HIV, IQR – interquartile range, NA – not available.

**Figure 2:**
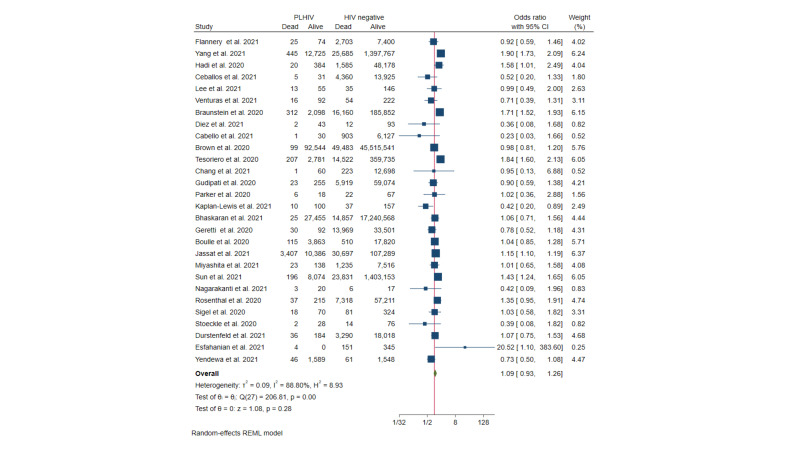
Forest plot showing the proportion of deaths among people living with HIV (PLHIV) and Human Immunodeficiency Virus (HIV)- seronegative patients.

Overall, the present systematic review included 168 531 PLHIV and 66 712 091 HIV-seronegative patients with SARS-CoV-2 infection. The number of deaths among PLHIV was of 5127, while 217 723 died among HIV-seronegative patients (**Table 1**). Accordingly, the meta-analysis showed no difference in the risk of death between PLHIV and HIV-seronegative patients (OR = 1.09; 95% CI = 0.93-1.26; *P* > 0.05) (**Figure 2**). However, a significant heterogeneity was found for this comparison (*I^2^* = 88.8%, *P* < 0.001).

**Table 1** also illustrates the risk of bias for studies included in the meta-analysis. According to the NOS, the quality score was calculated for the 26 retrospective studies and 2 prospective studies. All studies showed a low-to-moderate risk of bias, with a total score ranging from 4 to 7. Specifically, all studies were given a maximum of 3 stars out of 4 for selection category, 1 star out of 2 for comparability category, and 3 stars for the exposure/outcome category.

**Figure 3:**
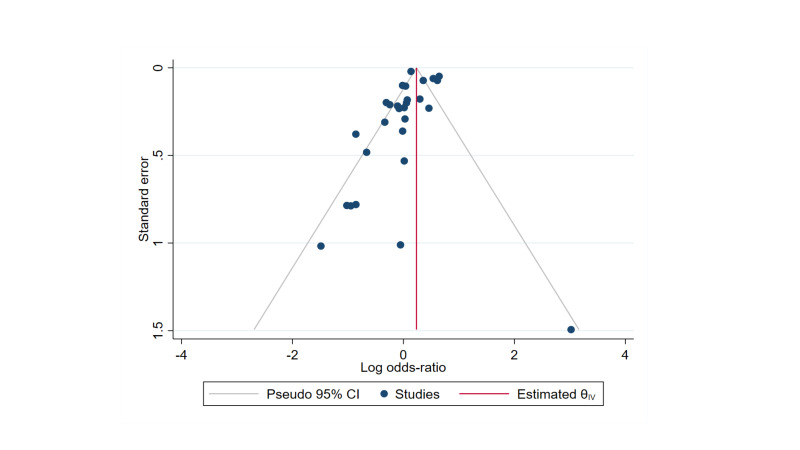
Funnel plot of studies included in the meta-analysis.

Moreover, visual inspection of funnel plot and Egger’s test were evaluated to assess the presence of publication bias for the studies included in the present meta-analysis. Interestingly, the funnel plot symmetry showed no evidence of publication bias for the risk death among PLHIV and HIV- seronegative people (**Figure 3**), which was also confirmed by the non-statistically significant Egger’s test (*P* > 0.05).

## DISCUSSION

In the current work, we evaluated the association between HIV infection and the risk of death of COVID-19 patients, by pooling data from 28 studies on 168 531 PLHIV and 66 712 091 HIV-seronegative individuals. To the best of our knowledge, this currently represents the most comprehensive meta-analysis on this topic. Our results showed no association between HIV infection and the risk of death among COVID-19 patients, although this finding was affected by significant heterogeneity between studies.

In the last years, persistent efforts have been made to evaluate the impact of SARS-CoV-2 infection and COVID-19 on vulnerable patients with previous comorbidities. Efforts that are certainly useful to identify those groups of people that need more attention and specific Public Health interventions. Although PLHIV constitute one of the most vulnerable groups, current evidence is still inconclusive about the effect of SARS-CoV-2 infection on the risk of death of these individuals [15,16,24-30]. The first attempt to disentangle the argument was made by Kouhpayeh and colleagues, who pooled data from 11 epidemiological studies. In particular, the authors reported that PLHIV had a 20% greater risk of death than their HIV seronegative counterpart [32]. This finding, however, has been later debunked by Danwang and colleagues, who carried out a meta-analysis of 23 epidemiological studies. In this case, in fact, the authors revealed that HIV infection was not associated with the risk of death and other adverse outcomes in patients with COVID-19 [31]. Our results, therefore, were in line with the current evidence that HIV infection does not affect the risk of death among COVID-19 patients.

However, some considerations should be made about the clinical status and preexisting chronic conditions that often characterize PLHIV. First of all, HIV infection could attenuate the immune response of these patients, increasing their risk of SARS-CoV-2 infection [10]. In addition, preexisting chronic conditions (eg, hypertension, diabetes, and cardiovascular diseases) could make PLHIV more prone to severe outcomes and mortality if infected with SARS-CoV-2 [50,58]. For both these reasons, many studies reported higher rates of hospitalization and death among PLHIV and SARS-CoV-2 infection [16,21,59]. From a pathological point of view, it has been reported that people with HIV and SARS-CoV-2 co-infection had higher levels of inflammatory markers, lymphopenia and lower CD4+ T cell counts [21,59,60]. These conditions could generate a severe inflammatory response to the SARS-CoV-2 infection, putting PLHIV at higher risk for the severe lymphopenia caused by COVID-19. For the sake of completeness, however, it should be also underlined that some studies proposed low levels of CD4 count and immunodeficiency of PLHIV as protective factors against the inflammatory response to SARS-CoV-2 infection [10]. These controversial opinions are also reflected by previous findings on the potential impact of HIV infection on COVID-19 symptoms and clinical course. While some studies reported worst symptoms among PLHIV, others did not reveal differences with HIV-seronegative people [61,62]. Therefore, further research is still needed to solve controversies about the risks associated with HIV infection in COVID-19 patients, taking into account clinical conditions of PLHIV and pathological events of the inflammatory response to SARS-CoV-2 infection.

The strength of our work was represented by the inclusion of a highest number of epidemiological studies than the previous meta-analyses on the same topic. However, our work had also some limitations that should be considered and discussed. First, there were both clinical and methodological differences (eg, study design, location, study population, etc.) that resulted in a significant heterogeneity between studies. For this reason, we applied the random effect model to calculate the pooled estimate. Second, most studies did not report information on age, sex, stages of HIV, symptoms, clinical characteristics, and access to medical care of included patients. This did not allow to adjust the analyses for unmeasured factors that could be associated with the progression of COVID-19 in PLHIV. Similarly, it was not possible to investigate biological mechanisms related to the HIV and SARS-CoV-2 co-infection [2,17,28]. Third, the majority of studies included in the meta-analysis were retrospective, representing a potential source of bias to be considered. Finally, the study protocol was not registered on the PROSPERO database and the search strategy was not conducted using MESH terms. We recognize that these are two of the main weaknesses of our study, but our choice was motivated by the urgent need of a comprehensive summary of findings on the risk of death associated with HIV and SARS-CoV-2 co-infection.

## CONCLUSIONS

In conclusion, our meta-analysis showed no difference in the risk of death among COVID-19 patients, when comparing PLHIV and HIV-seronegative individuals. Although this evidence was partially in line with previous findings, further research should be encouraged to better assess the impact of HIV and SARS-CoV-2 co-infection on clinical characteristics and prognosis of PLHIV.

## Additional material


Online Supplementary Document
